# *Sirdavidia*, an extraordinary new genus of Annonaceae from Gabon

**DOI:** 10.3897/phytokeys.46.8937

**Published:** 2015-02-04

**Authors:** Thomas L.P. Couvreur, Raoul Niangadouma, Bonaventure Sonké, Hervé Sauquet

**Affiliations:** 1 Institut de Recherche pour le Développement, UMR-DIADE, BP 64501, F-34394 Montpellier cedex 5, France.; 2 University of Yaoundé I, Higher Teacher’s Training College, Plant Systematic and Ecology Laboratory, P.O. Box 047, Yaoundé, Cameroon; 3 Naturalis Biodiversity Centre, Botany Section, Darwinweg 2, 2333 CR Leiden, The Netherlands; 4 National Herbarium of Gabon, R.D. 1135, Libreville, Gabon; 5 Missouri Botanical Garden, Africa & Madagascar Department, P.O. Box 299, St. Louis, Missouri 63166-0299, USA; 6 Université Libre de Bruxelles, Herbarium et Bibliothèque de Botanique africaine, CP 169, Av. F. Roosevelt 50, B-1050, Brussels, Belgium; 7 Université Paris-Sud, Laboratoire Écologie, Systématique, Évolution, CNRS UMR 8079, 91405 Orsay, France

**Keywords:** Piptostigmateae, Monts de Cristal, buzz pollination, vicariance, Annonaceae, Central Africa, Magnoliidae

## Abstract

A distinctive new monotypic genus from Gabon is described in the tropical plant family Annonaceae: *Sirdavidia*, in honor to Sir David Attenborough. Molecular phylogenetic analyses confirm that *Sirdavidia*, which is very distinct from a morphological standpoint, is not nested in any existing genus of Annonaceae and belongs to tribe Piptostigmateae (subfamily Malmeoideae), which now contains a total of six genera. The genus is characterized by long acuminate leaves, fully reflexed red petals, 16–19 bright yellow, loosely arranged stamens forming a cone, and a single carpel topped by a conspicuous stigma. With just three known collections, a preliminary IUCN conservation status assessment is provided as “endangered” as well as a distribution map. The discovery of *Sirdavidia* is remarkable at several levels. First, it was collected near the road in one of the botanically best-known regions of Gabon: Monts de Cristal National Park. Second, its sister group is the genus *Mwasumbia*, also monotypic, endemic to a small area in a forest in Tanzania, some 3000 km away. Finally, the floral morphology is highly suggestive of a buzz pollination syndrome. If confirmed, this would be the first documentation of such a pollination syndrome in Magnoliidae and early-diverging angiosperms in general.

## Abstract (fr)

Un nouveau genre monotypique du Gabon est décrit dans la famille tropicale des Annonaceae: *Sirdavidia*, en honneur à Sir David Attenborough. Des analyses phylogénétiques confirment que *Sirdavidia*, caractérisé par une morphologie unique, n’appartient à aucun genre connu d’Annonaceae et se place au sein de la tribu des Piptostigmateae (dans la sous-famille des Malmeoideae), laquelle compte désormais un total de six genres. Le genre est caractérisé par des feuilles avec un long acumen, des pétales rouges réfléchis, 16–19 étamines jaunes qui forment un cône lâche et un carpelle surmonté d’un stigmate bien visible. Avec seulement trois récoltes connues, un statut de conservation préliminaire de “endangered” est proposé sur la base des critères de l’UICN. Une carte de distribution est également fournie. La découverte de *Sirdavidia* est remarquable pour plusieurs raisons. Tout d’abord le nouveau genre a été collecté près de la route dans l’une des régions les plus connues botaniquement du Gabon: le Parc National des Monts de Cristal. Ensuite, son groupe-frère est le genre, *Mwasumbia*, également monotypique, endémique d’une petite région en Tanzanie à plus de 3000 km. Enfin, les caractères floraux suggèrent un syndrome de pollinisation appélé « buzz pollination». Si cela se confirme, ce sera la première documentation de ce type de syndrome pour les Magnoliidae et les Angiospermes basales en général.

## Introduction

The Central African country of Gabon is merely 270 000 km² in size, but is home to an incredible botanical diversity (Sosef et al. 2006). Around 82% of its territory is covered with tropical rain forest and with around 5000 vascular plant species, Gabon is an important center of plant biodiversity in Central Africa (Sosef et al. 2006). The country is botanically one of the best known in the region (Sosef et al. 2006; Wieringa and Sosef 2011) with several parts of its territory well inventoried, such as the Monts de Cristal area (Wieringa and Sosef 2011).

Annonaceae (Magnoliidae) is a pantropical flowering plant family of trees, shrubs and lianas. With around 2500 species (Chatrou et al. 2012; Couvreur et al. 2011) it is one of the most diverse plant families in tropical rain forests, and the largest in order Magnoliales (Haywood et al. 2009). Recently a new phylogenetic classification of the family recognizes four subfamilies and 14 tribes (Chatrou et al. 2012). This was followed by a scratchpad website (World Annonaceae; Couvreur 2014b) documenting Annonaceae diversity and taxonomy worldwide. Taxonomic understanding of African Annonaceae has been increasing since the publication of “Flore du Gabon, Annonaceae, volume 16” (Le Thomas 1969) more than 40 years ago (Botermans et al. 2011; Couvreur 2009; Couvreur 2014a; Deroin and Luke 2005; Fero et al. 2014; Versteegh and Sosef 2007). As a consequence, several new species and a new genus have been described in Africa these past years, mainly from Tanzania (Couvreur et al. 2006; Couvreur et al. 2009; Johnson et al. 1999; Luke and Deroin 2005; Marshall et al. in press). New species, however, from Central Africa, and in particular Gabon, have been rarer with only a few such descriptions (Jongkind 2002).

A probable new genus of Annonaceae was collected during field work in Monts de Cristal National Park, as part of a larger field trip focusing on the study Magnoliidae floral diversity. The objective of this paper was to confirm its status as a new species and its classification in a new genus. The taxon was first seen near the Kinguélé dam, and further prospection in the area revealed several individuals. It is the unusual floral structure of this species for Annonaceae that led us to suspect it might represent a new taxon and to undertake both a phylogenetic analysis and more thorough morphological observations. As we show here, interesting tropical taxa unknown to science can still be discovered in places even considered to be well known botanically.

## Material and methods

Herbarium, alcohol and photographic materials were used to produce the descriptions. In order to identify other specimens of this new genus, we looked at all undetermined Annonaceae specimens in the herbaria located at BR, BRLU, LBV, P and YA (herbarium acronyms according to Thiers 2012). We also looked at sterile plot specimens of Annonaceae for Gabon held at BRLU. The conservation status was assessed by calculating the extent of occurrence (EOO) and the area of occupancy (AOO) using the GeoCAT tool (Bachman et al. 2011) and applying the IUCN Red List Category criteria (Standards-and-Petitions-Working-Group 2006).

A preliminary phylogenetic analysis indicated that the new taxon was nested in tribe Piptostigmateae of the Malmeoideae subfamily. Therefore, the data matrix of Couvreur et al. (2009) was used to undertake the analyses. The matrix was based on two plastid markers (*rbcL* and *trnL* intron / *trnL-trnF* spacer) and contains 35 out of the 47 genera of Malmeoideae, representing all major lineages. Representative species from all other subfamilies were also sampled: Anaxagoreoideae (1 genus), Ambavioideae (2 genera out of 8), Annonoideae (17 genera out of 50). *Eupomatia bennettii* (Eupomatiaceae) was chosen as the outgroup (Massoni et al. 2014; Sauquet et al. 2003). All six genera currently recognized in Piptostigmateae were sampled. Sampling within genera was restricted to one species in the Annonoideae and Ambavioideae, and varied from one to two species in the Malmeoideae.

DNA extractions of silicagel-dried leaf samples from two individuals of *Sirdavidia solannona* Couvreur & Sauquet were performed using a DNeasy Plant Mini Kit (Qiagen, Valencia, CA). The universal primers C/D and E/F (Taberlet et al. 1991) were used to amplify and sequence the *trnL* intron and *trnL-trnF* spacer. The *rbcL* marker was amplified using two primer combinations, 1F/724R and 636F/1460R (Fay et al. 1998). PCR amplifications were conducted using the FailSafe kit with Premix E (Epicentre, Madison, WI), according to manufacturer’s instructions and by adding 0.5 U of *Taq* DNA polymerase (Promega, Madison, WI) in a total volume of 50 μL. The PCR program was as follows: 35 thermal cycles at 94 °C for 1 min, 50–55 °C for 50 s, 72 °C for 50 s and a final extension at 72 °C for 3 min. Sequencing was performed at Macrogen (The Netherlands). Sequences were edited using Geneious 1.5.6 (Drummond et al. 2010) and manually aligned in the PAUP* text editor (version 4.10b; Swofford 2002). Gaps were coded following the simple coding model of Simmons and Ochoterena (2000). Microsatellites and ambiguously aligned regions (in the *trnL* intron and *trnL-trnF* spacer) were excluded from the analyses.

Maximum Parsimony (MP) analyses were performed using PAUP* (version 4.10b; Swofford 2002). Heuristic searches were performed with 100 random taxon addition sequence iterations, saving 100 trees at each iteration, with tree bisection-reconnection branch swapping. Relative support for each node was assessed by performing 1000 bootstrap (BS) replicates (Felsenstein 1985) with TBR branch swapping (20 random addition sequences, saving 20 trees per replicate).

Maximum likelihood analyses were conducted using RAxML version 7.2.7 (Stamatakis 2006) on the CIPRES portal teragrid (Miller et al. 2009). ML bootstrap analyses and the inference of the optimal tree were conducted simultaneously. The optimal tree was inferred using a GTR+Γ model, whereas a similar yet more computationally efficient model (GTR+CAT) was employed for the 1000 bootstrap iterations (Stamatakis et al. 2008).

## Results

All Genbank numbers used can be found in Couvreur et al. (2009). The Genbank numbers of the newly sequenced *Sirdavidia solannona* are: Couvreur 596, trnLF: KP144079; rbcL: KP144081; Couvreur 597, trnLF: KP144080; rbcL: KP144082.

**Figure 1.**
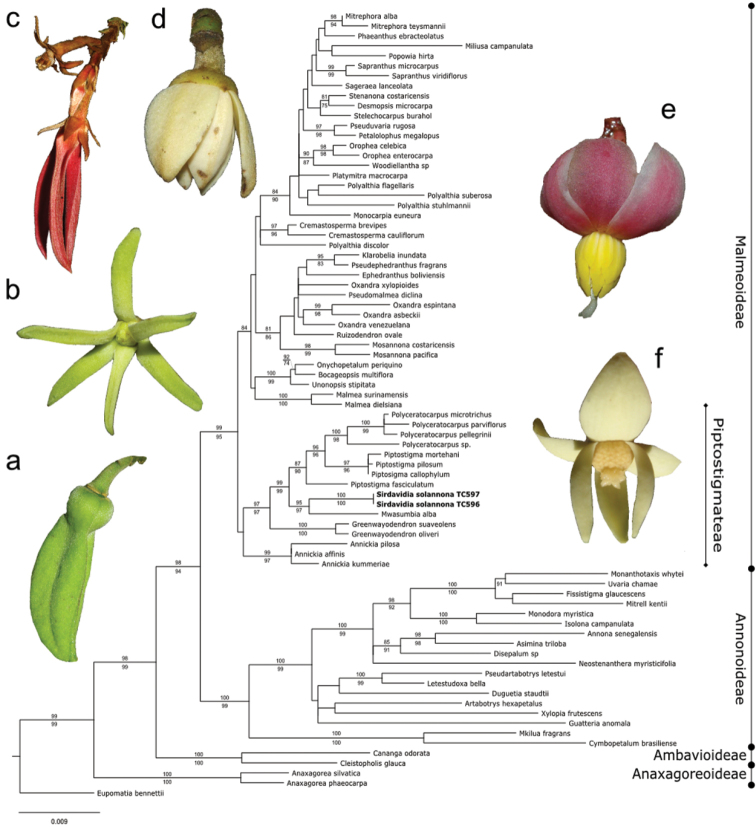
Maximum likelihood tree with support values indicated on branches (ML bootstrap above; MP bootstrap below). Flower morphology of the genera in the Piptostigmateae tribe. **a** *Annickia affinis* (Exell) Versteegh & Sosef **b** *Greenwayodendron suaveolens* (Engl. & Diels) Verdc **c** *Piptostigma multinervium* Engl. & Diels **d** *Polyceratocarpus parviflorus* (Baker) Ghesq **e** *Sirdavidia solannona* **f** *Mwasumbia alba*. Photos: TLP Couvreur. Note: there is some confusion around the proper identification of the accession Lugas 111 (*Woodiellantha* sp in this study).

Both markers represented 2669 total characters, 187 of which were excluded because of ambiguity in the alignment and 407 (16.5%) were parsimony informative. Both MP and ML phylogenetic analyses led to the same topology, with similar levels of support (Fig. 1). In these trees, *Sirdavidia solannona* is nested in Piptostigmateae with strong support and is sister to *Mwasumbia alba* Couvreur & Johnson (MP-BS = 95%; ML-BS = 97%).

## Taxonomic description

### Sirdavidia

Taxon classificationPlantaeMagnolialesAnnonaceae

Couvreur & Sauquet gen. nov.

urn:lsid:ipni.org:names:77145065-1

#### Diagnosis.

Genus with *Solanum*-like flowers, inflorescences axillary or cauliflorous, sepals valvate, petals valvate, subequal, recurved at anthesis, red; stamens bright yellow; carpel single; monocarp sessile, placentation lateral, ovules uniseriate.

#### Type species.

*Sirdavidia solannona* Couvreur & Sauquet.

Small trees with distichous, simple pinnately veined leaves with an entire margin and reticulate third-order venation. Species androdioecious (?) (flowers unisexual staminate or bisexual). Inflorescences one to three-flowered, axillary on old branches or at base of trunk, with one to three short sympodial rachilla. Flowers actinomorphic. Perianth of 9 free tepals in 3 alternate, valvate whorls of 3 each, differentiated in outer tepals (sepals) and middle and inner tepals (petals). Petals similar (subequal in length), spreading horizontally or reflexed at anthesis. Stamens 16-19, free, basifixed with a very short filament. Anthers introrse, probably opening by two longitudinal slits, connectives tongue shaped, yellow. Carpel one, densely pubescent, stigma cylindrical coiled, ovules 7–10, uniseriate. Monocarp sessile, cylindrical densely pubescent.

**Figure 2.**
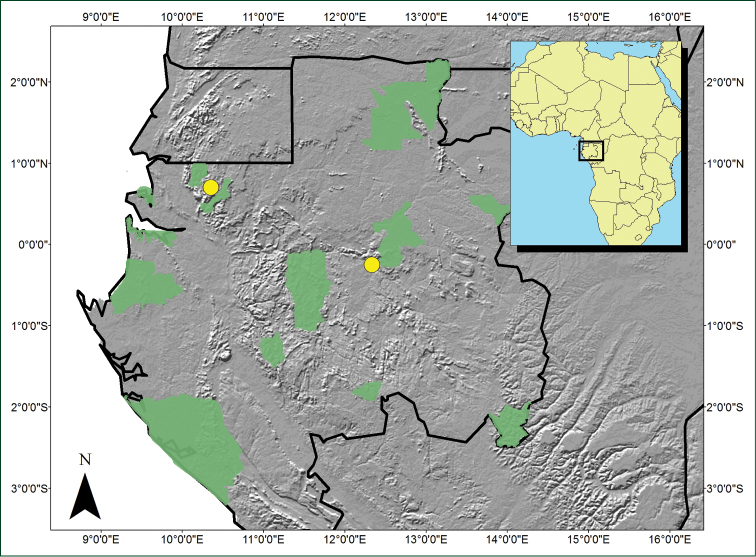
Distribution map of *Sirdavidia solannona*. Grey scale color shows elevation variation; Gabonese National Parks highlighted in green.

A single species only known to Gabon (Fig. 2).

#### Etymology.

We dedicate this new genus to Sir David Attenborough, British broadcaster and naturalist, in honor of his lifelong dedication to nature, conservation, evolution and natural history programs. His passion for nature have influenced and inspired a generation of biologists and naturalists, including the first and senior authors of this paper.

### Sirdavidia solannona

Taxon classificationPlantaeMagnolialesAnnonaceae

Couvreur & Sauquet sp. nov.

urn:lsid:ipni.org:names:77145066-1

#### Type.

Gabon, Estuaire, Monts de Cristal, near first bridge after Kinguele, 0°46'66"N, 10°27'81"E, *T.L.P. Couvreur 596*, 15 Nov 2013, Fl. & Fr., holotype: WAG!; isotypes: LBV!, P!, YA!.

**Figure 3.**
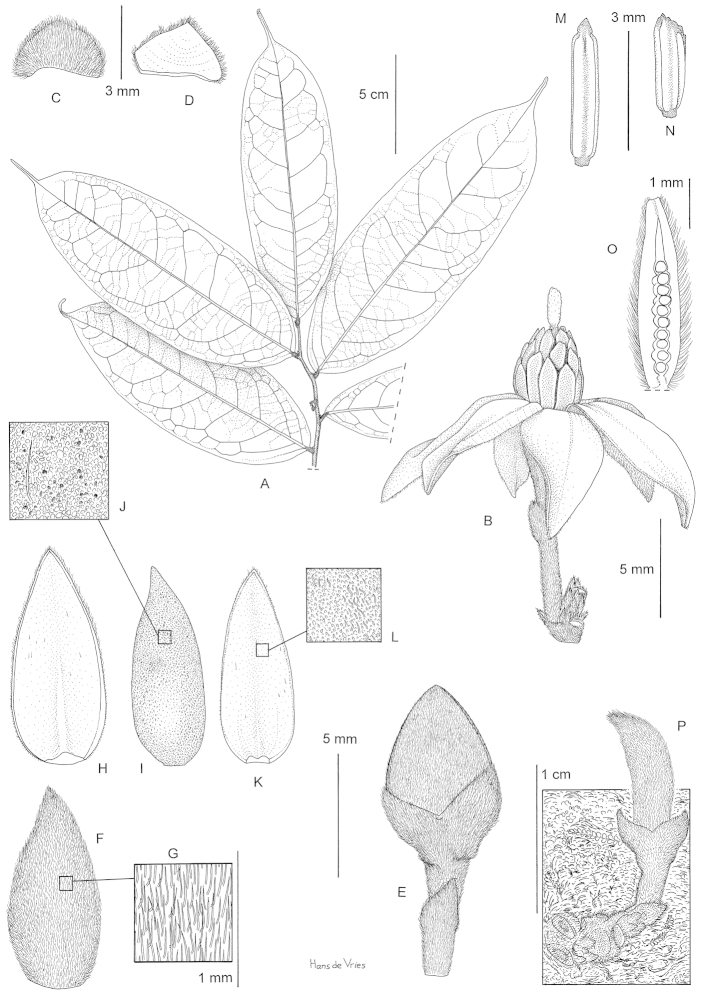
Illustration of *Sirdavidia solannona* Couvreur & Sauquet. **A** Flowering branch (flower bud just above second leaf from the bottom) **B** Flower **C** One sepal, outer side view **D** One sepal, inner side view **E** Flower bud **F** Outer petal, outer side view **G** detail of pubescence of outer petal, outer side **H** Outer petal, inner side view **I** Inner petal, outer side view **J** detail of pubescence of inner petal, outer side **K** Inner petal, inner side view **L** detail of pubescence of inner petal, inner side **M** Stamen from inner whorl **N** stamen from outer whorl **O** Longitudinal section of carpel showing uniseriate row of ovules (stigma missing) **P** detail of young fruit. Drawing by Hans de Vries based on Couvreur 596 and Couvreur 597.

**Figure 4.**
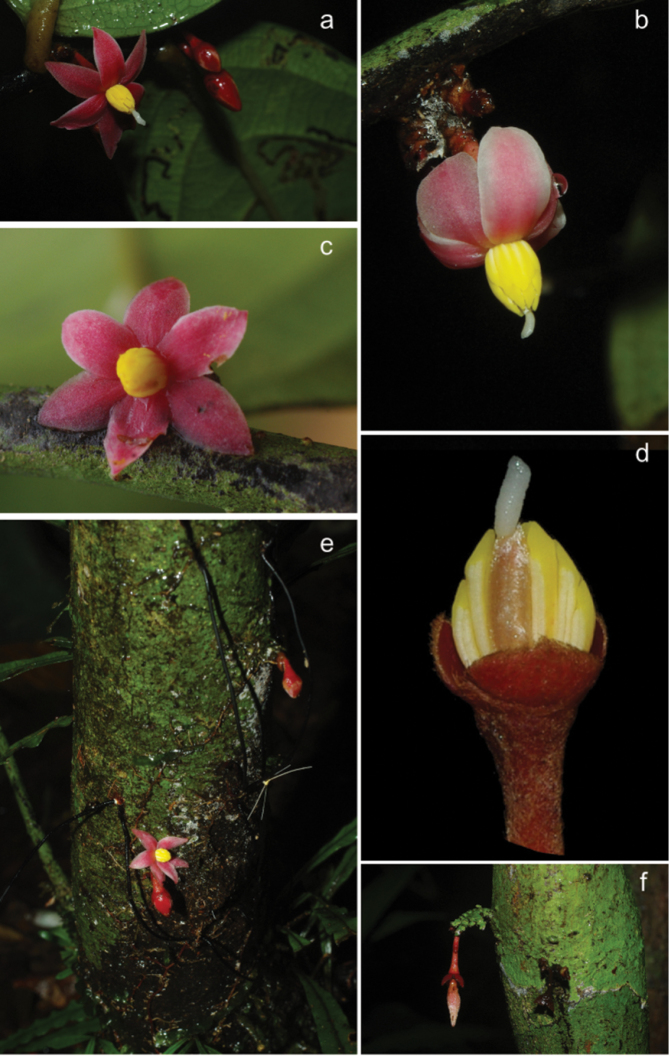
*Sirdavidia solannona*. **a** Opened flower and flower buds (Couvreur 596) **b** Flower with recurved petals at anthesis (Couvreur 596) **c** Staminate flower (Couvreur 597) **d** Flower with all petals and part of the stamens removed, showing the silvery aspect of the carpel and the long stigma (Couvreur 596) **e** Cauliflorous flower and flower bud (Couvreur 596) **f** Young fruit, cauliflorous (Couvreur 596). Photos: TLP Couvreur.

Tree 4–6 m tall, 2 to 4 cm in diameter at breast hight (d.b.h.), bark dark brown with patches of green, old branches black, glabrous, young branches black, sometimes pubescent. Leaves distichous, simple, entire, pinnately veined. Petiole 3–4 mm long, 2–3 mm in diameter, glabrous or sparsely pubescent when young, slightly grooved on top, leaf lamina inserted on top. Lamina 20–26 cm long, 4.5 to 9 cm wide, length:width ratio 2.5 to 4.5, narrowly elliptic to elliptic to narrowly ovate to ovate, apex long acuminate, acumen 2–3 cm long, base obtuse, coriaceous, young sparsely pubescent to glabrous above, glabrous below, old leaves glabrous above and below, mid rib sunken above, sparsely pubescent when young below, glabrous above, glabrous above and below when old, secondary veins 9–12 pairs. Inflorescences axillary, on old branches and cauliflorous towards the base of the trunk. Sympodial rachis up to 6 mm long, but sometimes up to 1.5 cm long, densely covered with short appressed hairs, with 0–10 minute densely packed lower bracts densely pubescent brown. Flowering pedicels 2 to 10 mm long, densely covered with short appressed hairs, red, upper bract inserted at base or up to ½ of pedicel, covered with short appressed hairs, red. Flowers actinomorphic, bisexual or unisexual staminate (androdioecious), with 9 tepals in total, differentiated in one whorl of 3 sepals and 2 whorls of 3 petals, all alternate. Sepals 2–3 mm long, 1.5–2 mm wide, length:width ratio 1.5, ovate, valvate, apex acute, base truncate, densely covered with short appressed hairs outside, glabrous inside, red. Outer petals 4–10 mm long, 2.5 to 5 mm wide, length:width ratio 2 to 2.5, elliptic, apex acute, base truncate, densely pubescent with appressed hairs outside, densely pubescent with short tomentose hairs inside, deep red. Inner petals 4–9 mm long, 2–4 mm wide, length:width ratio=2 to 2.5, elliptic, apex acute, base truncate, densely pubescent with short tomentose hairs outside, densely pubescent with short tomentose hairs inside along margins, glabrous towards center, deep red. Petals spreading horizontally or recurving backwards at anthesis. In staminate and bisexual flowers, stamens 16–19, 3–4 mm long, outer ones shorter than inner ones, filament shorter than 0.2 mm, narrow, connective umbonate (tongue shaped), glabrous, bright yellow. Anthers introrse, probably opening by two longitudinal slits. In bisexual flowers, carpel one, 4–5 mm long, ca. 1 mm wide, densely pubescent with silvery long appressed hairs, ovules uniseriate, 7–10, stigma cylindrical coiled, 2–3 mm long, sparsely pubescent towards the top, white cream. Mature fruits not seen, young fruiting pedicel 6 mm long, densely pubescent with appressed hairs. Young monocarp cylindrical, densely pubescent with silvery appressed hairs. Seeds not seen. (Figs 3 and 4)

#### Phenology.

Flowers collected in April and November, young fruits collected in November.

#### Distribution and habitat.

*Sirdavidia* is endemic to Gabon, with three known collections: two near the Kinguele dam in the Monts de Cristal National Park, Mbé sector, and one south of the Ivindo National Park (Fig. 2). Floristic comparisons in Gabon emphasize that the Monts de Cristal flora has a high resemblance with many other areas across Gabon, including the Ivindo NP region (Wieringa and Sosef 2011). Thus it is not unusual to find species occurring in Monts de Cristal and elsewhere in the county. *Sirdavidia* grows in the understory of mature to old secondary rain forests around 300–600 m, near rivers or on inundated soils.

#### Preliminary conservation assessment.

Endangered [EN B1ac]. Two localities in Gabon are known for this species: Monts de Cristal N.P. and south of the Ivindo N.P. The population found in Kinguele (Monts de Cristal) was close to the road and several (around 10) individuals were seen. We also looked for this species in other parts of the Park (around Tchimbélé) and did not see it again. The herbarium specimen collected from Ivindo indicates “en peuplement” (in population) suggesting that several individuals were seen. However, the coordinates on the herbarium sheet place this collection outside the national park. The Area of occupancy (AOO) is 12,000 km² and the Extent of occurrence (EOO) is 6.2 km², suggesting a very restricted overall distribution. We thus suggest a status of endangered given that only a handful of individuals have been seen and that these populations are quite close to disturbances.

#### Etymology.

The species name epiteth highlights the striking resemblance with flowers of some species of *Solanum*, an unusual and new feature for a flower of Annonaceae.

#### Note.

The androdioecious nature of *Sirdavidia solannona* has yet to be properly confirmed. We only saw two individuals one of which appeared to have only staminate flowers. Because other members of the tribe Piptostigmateae are known to have this condition, it would not be surprising.

#### Paratypes.

Gabon: **Estuaire**, Monts de Cristal National Park, near first bridge after Kinguele, 0°46'64"N, 10°27'80"E, *T.L.P. Couvreur 597*, Fl., 15 Nov 2013, Fl. & Fr. (LBV!, P!, WAG!, YA!); **Ougoué-Ivindo**, Ivindo National Park, camp elephant, *A. Moungazi 1544*, Fl., 10 Avr 204 (BR!, LBV, WAG).

**Table 1.** Morphological characters of the six genera found in tribe Piptostigmateae. Modified from Couvreur et al. (2009). *Piptostigma* is represented by two columns because it is paraphyletic (Fig. 1).

Genus	*Annickia*	*Greenwayodendron*	*Mwasumbia*	*Sirdavidia*	*Piptostigma fasciculatum*	*Piptostigma*	*Polyceratocarpus*
Character							
Species diversity/distribution	8 / West and Central Africa, 1 species in East Africa	2 / West and Central Africa	1 / Tanzania	1 /Gabon	1 / Central Africa	~14 / Central and West Africa	8 / West and Central Africa, 2 species in East Africa
Tertiary venation	intermediate	reticulate	intermediate	reticulate	parallel	parallel	parallel
Inflorescence position	terminal	terminal	axillary	axillary, cauliflorous	axillary	axillary, cauliflorous	axillary, cauliflorous
Sex distribution	bisexual	androdioecious	bisexual (?)	androdioecious (?)	bisexual	bisexual	androdioecious
Sepal aestivation	valvate	imbricate	imbricate	valvate	valvate	valvate	valvate
Petal number	3	6	6	6	6	6	6
Petal disposition	Upright, appressed forming a pollination chamber	Spreading horizontally, no pollination chamber	Outer petals recurved backwards, inner petals erect upwards, no pollination chamber	Recurving backwards to spreading horizontally, no pollination chamber	Pendulous, no pollination chamber	Upright, appressed forming a pollination chamber	Outer petals recurved backwards or erect upwards, inner petals erect upwards, pollination chamber possible
Petal relative length	outer absent	outer=inner	outer=inner	outer=inner	outer<inner	outer<inner	outer=inner
Torus (stamen portion)	flat/conical	flat/conical	short cylindrical	short cylindrical	short cylindrical	short cylindrical	short cylindrical
Torus (carpel portion)	flat/convex	flat/convex	concave	concave	concave	concave	concave
Apex of connective	discoid/tongue-shaped	discoid/tongue-shaped	discoid	discoid/tongue-shaped	discoid	discoid	discoid
Nr of carpels	numerous	13–20	4	1	4	3–14	2–20
Number of ovules per carpel	1	2	5–8	7	~ 18	6–10	20–30
Ovule arrangement	basal	1-seriate lateral	1-seriate lateral	1-seriate lateral	2-seriate	2 or 1-seriate lateral	2-seriate lateral
Monocarps	stipitate	stipitate	sessile	sessile	sessile	sessile	sessile

## Discussion

### Molecular and morphological characterization of *Sirdavidia*

The molecular phylogenetic analyses presented here confirms that *Sirdavidia solannona* belongs to tribe Piptostigmateae, which now contains a total of six accepted genera (though *Piptostigma* is paraphyletic, Couvreur et al. 2009). This new taxon was found to be sister with strong support to the monotypic East African genus *Mwasumbia* Couvreur & Johnson (see below, Fig. 1). The two genera, together with *Polyceratocarpus* Engl. & Diels and *Piptostigma* Oliv., form a strongly supported clade (Fig. 1), referred to here as the SMPP clade.

*Sirdavidia* differs morphologically from *Mwasumbia* in several important respects, warranting its status as a new species and a new genus. Tertiary venation is a useful character for distinguishing genera within the tribe Piptostigmateae (Couvreur et al. 2009) and contains useful phylogenetic information at the family level (Doyle and Le Thomas 1996). All three major tertiary venation types in Annonaceae (reticulate, parallel and intermediate between the two first ones) are found in Piptostigmateae (Table 1). Parallel tertiary venation occurs in both *Piptostigma* and most *Polyceratocarpus* species whereas *Mwasumbia* has an intermediate tertiary venation. In contrast, *Sirdavidia* is characterized by a reticulate tertiary venation and in this sense resembles *Greenwayodendron* (Table 1). This type of venation appears to be rare in Annonaceae and was reconstructed as being ancestral for the family as a whole (Doyle and Le Thomas 1996). Sepal aestivation in *Sirdavidia* is valvate like in most other genera in Piptostigmateae except for *Greenwayodendron* and *Mwasumbia* which both have an imbricate aestivation (Table 1). Aestivation was considered an important character to separate genera in Annonaceae (Chatrou et al. 2012), but phylogenetic studies have underlined its homoplastic nature (Couvreur et al. 2008c; Doyle and Le Thomas 1996). The presentation of petals at anthesis is also very different between *Sirdavidia* and *Mwasumbia*. In *Mwasumbia*, the outer petals are reflexed, whereas the inner petals are pendulous and sometimes connivent at the tips. A similar configuration is observed in some species of *Polyceratocarpus* (Marshall et al. in press). In contrast, the petals in *Sirdavidia* are horizontally spreading to highly reflexed at anthesis, a condition not found in any other genera within Piptostigmateae (Table 1, Figs 1e, 3B, 4a, b,c ). Stamen number and connective shape have played an important part in Annonaceae classification (Chatrou et al. 2012). In *Sirdavidia* they are very distinct to those in *Mwasumbia*. *Sirdavidia* has 16-19 stamens with, in general, a tongue shaped connective apex (Fig. 3M), while *Mwasumbia* has numerous stamens (more than 30) with a discoid or flat connective apex (Figure 3L of Couvreur et al. 2009).

We suggest that the morphological differences outlined above are sufficient to erect a new species for *Sirdavidia solannona* and also justify the creation of a new genus, distinct from *Mwasumbia*, given the morphological characters that discriminate among genera of Annonaceae in general (Chatrou et al. 2012; Le Thomas 1969).

### Morphological similarities and differences of *Sirdavidia* with Piptostigmateae genera

Table 1 summarizes the morphological similarities and differences of *Sirdavidia* with the other genera within Piptostigmateae. Two characters appear important for delimiting the SMPP clade:

– Inflorescence position: Within Piptostigmateae both terminal or axillary types of inflorescences can be found (Fries 1959). In *Sirdavidia*, inflorescences are axillary, a character also found in *Mwasumbia*, *Piptostigma* and *Polyceratocarpus* (Table 1) confirming it as a good synapomorphy for the SMPP clade within Piptostigmateae. The position of inflorescences has also previously been recognized as a good character for separating genera in Annonaceae (Chatrou et al. 2000).

– Monocarp base: Another synapomorphy for the SMPP clade is the sessile monocarps. In contrast, *Annickia* Setten & Maas and *Greenwayodendron* Verdc. have monocarps with conspicuous stipes. Even though immature fruits were only observed for *Sirdavidia* to date, it is clear that the single monocarp is sessile (Fig. 3P; Fig. 4f). Although this character was previously thought to contain little taxonomic information, it has proven useful in delimitating other African tribes of Annonaceae such as Monodoroideae (Chatrou et al. 2012; Couvreur et al. 2008c).

Other characters appear to have little taxonomic use but are interesting as they underline the important floral morphological variability within Piptostigmateae.

– Androdioecy in Annonaceae is not unusual (van Heusden 1992). Within tribe Piptostigmateae two other genera are documented as being androdioecious: *Greenwayodendron* and *Polyceratocarpus* (Couvreur et al. 2009). However, with only two individuals seen, it is difficult to conclude precisely on the type of reproductive strategy for *Sirdavidia*.

– *Sirdavidia* is unique within the tribe in having a single carpel, a feature found in only 10% of Annonaceae (Deroin 1991). Other genera such as *Sanrafaelia* Verdc., *Dielsiothamnus* R.E.Fr., and *Tridimeris* Baill. are also monocarpellate (van Heusden 1992; Verdcourt 1996).

### A fascinating new genus

*Sirdavidia* is fascinating at a number of other different levels. First, it had remained undescribed until now, even though it occurred in a well known and well inventoried region of Gabon; second, its closest relative is another monotypic genus restricted to Tanzania, some 3000 km away; and, finally, it could be the first documentation of a buzz pollination syndrome in Magnoliidae.

### A hidden genus

*Sirdavidia* was discovered in the Monts de Cristal National Park (N.P.), one of the most species-rich and botanically best collected regions in Gabon (Wieringa and Sosef 2011). Moreover, it was collected just a few meters from the main road that connects Kinguele to Tchimbele. This discovery suggests that there may still be a number of undescribed species and genera in this region and thus might not be as well collected as suggested. Interestingly, the small population was located near a recent botanical inventory of the Monts de Cristal (Sunderland et al. 2004). Because it is a tree that so far has been observed to be smaller than 6 m and is less than 10 cm in diameter, it is likely that it was not collected during the inventories (in general such inventories only focus on trees with a diameter larger than 10 cm). This underlines the importance of collecting woody individuals with a diameter less than 10 cm when undertaking inventory plots (Gentry and Dodson 1987; Kenfack et al. 2007). An alternative explanation is that because it superficially doesn’t look like an Annonaceae flower, putative collections might have been identified under different plant families.

### An incredible disjunction

The closest relative to *Sirdavidia* is another recently described monotypic genus, *Mwasumbia* (Couvreur et al. 2009). This rain forest genus is endemic to a small locality in the east African forests of Tanzania, corresponding to a biogeographic disjunction of ca. 3000 km with *Sirdavidia*. East West/Central disjunctions between rain forest restricted species are a common feature in African plants (Burgess et al. 2007; Couvreur et al. 2008b; Lovett 1993). However, this might represent an extreme disjunction between two locally restricted rain forest monotypic genera on opposite sides of Africa. Several disjunctions between East and West/Central African Annonaceae have been dated to occur at significantly different periods of increased aridity suggesting a repeated continental scale fragmentation of a once pantropical rain forest (Couvreur et al. 2008b). These two genera provide another example of the role of this vicariant pattern in generating endemicity (both faunistic and floristic) across tropical Africa (Couvreur et al. 2008b; Tolley et al. 2013). It will be interesting to estimate the temporal origin of this disjunction in order to measure the evolutionary time these two species represent and to determine whether their splitting coincides with those of other East/West African disjunctions in Annonaceae and other tropical plant families.

### A possible new pollination syndrome type for Magnollideae

Though the flower has all the structural characters of a typical Annonaceae flower (3 sepals, 2 whorls of 3 petals), the overall aspect is very unusual, resembling flowers of some species of *Solanum* L. (Solanaceae). The morphological appearance is strongly suggestive of a special type of pollination syndrome referred to as buzz pollination syndrome. Buzz pollination relies mostly on sonicating bees that use vibrations to extract pollen from the anthers (De Luca and Vallejo-Marín 2013). The flowers of *Sirdavidia* are characterized by several traits typically associated with the evolution of buzz pollination.

Reflexed petals. Most buzz pollinated flowers show strongly reflexed petals exposing the stamens and the carpels. Reflexed petals are quite common in Annonaceae, occurring in a number of genera such as *Uvaria* and *Isolona*.

Stamens: The stamens of *Sirdavidia* are bright yellow, a color known to attract bees (De Luca and Vallejo-Marín 2013). Such a color is unusual for Annonaceae, at least in Africa. In general, stamens are pale in color, varying between red, green and yellow. In addition, typical stamens of Annonaceae are never fully exposed as they are in *Sirdavidia*, being generally tightly packed together and appressed by the petals. Non appressed stamens by the petals are also found in the sister genus *Mwasumbia* (Couvreur et al. 2009). In *Sirdavidia* the stamens form a “cone” of loosely arranged stamens (relative to other Annonaceae species) around the single carpel, a feature also found in buzz pollinated *Solanum*-type flowers (De Luca and Vallejo-Marín 2013).

Anthers. In typical buzz pollinated flowers, the anthers generally have apical pores or short slits that release the pollen grains during vibration. However, non-poricidal anthers have also been linked with buzz pollination in a number of other genera (Buchmann 1985; de Oliveira and Sazima 1990). Based on macromorphological observations, no evidence of apical pores can be seen in *Sirdavidia*, which would thus rather represent a case of non-poricidal buzz pollination. It is possible that the structural longitudinal slits we have observed only effectively dehisce apically, thus functioning as short apical slits. However, detailed anatomical observations will have to confirm this. In addition, buzz pollination will only be effective if pollen grains are very small and extremely numerous (dust like). To date, we have no information about the size and quantity of pollen grains in *Sirdavidia*. Pollen in Annonaceae is generally large in size compared to other angiosperms (Doyle and Le Thomas 2012). Pollen grains in the sister genus *Mwasumbia* are monads and were measured to be ca. 59 µm in length for the polar axis, which ranks as a medium-sized pollen grain in Annonaceae (Couvreur et al. 2008a; Doyle and Le Thomas 2012). However, pollen size is highly homoplasic in Annonaceae (Doyle and Le Thomas 2012), and thus it is hard to speculate on the size of the pollen grains in *Sirdavidia*.

Long stigma. The conspicuous stigma sticking out of the stamens in *Sirdavidia* is also a typical feature of buzz pollinated flowers (De Luca and Vallejo-Marín 2013). The stigma rubs against the abdomen of the visiting bee allowing the potential pollination.

Annonaceae flowers are visited by a large variety of insects (Saunders 2012; Silberbauer-Gottsberger et al. 2003) such as beetles, thrips flies and even cockroaches, suggesting a large array of pollination systems. In contrast, bees have rarely been documented to pollinate or visit Annonaceae flowers (Silberbauer-Gottsberger et al. 2003). Bee pollination is suspected in *Unonopsis guatterioides* (A.DC.) R.E.Fr. and *Uvaria concava* Teijsm. & Binn. However, flowers of these two species are apparently not buzz pollinated, and are very different in morphology than those of *Sirdavidia*. Additional field observations will be required to determine whether *Sirdavidia* is indeed buzz pollinated. In addition to observations of pollinator behavior, it would be particularly interesting to learn more about the floral biology of this species. Indeed, nearly all early-diverging angiosperms (including Annonaceae) are protogynous, a feature commonly associated with wind, beetle, fly, and thrips pollination, whereas the remaining angiosperms are predominantly protandrous and bee or butterfly pollinated (Endress 2010). Therefore, one would expect that *Sirdavidia* might have shifted away from protogyny to allow effective buzz pollination by pollen collecting bees. If the buzz pollination syndrome was to be confirmed for *Sirdavidia*, it would be the first record in Annonaceae and for Magnoliidae and early-diverging angiosperms in general (Endress 2001). In any case, this represents the first “*Solanum*-type” flower described in Magnoliidae to date (De Luca and Vallejo-Marín 2013; Endress 2001).

## Supplementary Material

XML Treatment for Sirdavidia

XML Treatment for Sirdavidia solannona
